# William Richard Huggard

**Published:** 1911-12

**Authors:** 


### Obituary Notice.

### WILLIAM RICHARD HUGGARD, M.D., LL.D.

*The Davos Courier* of Oct. 13th and 20th, 1911, gives us full particulars of the life and death of His Majesty's Consul at Davos, a physician of rare and refined personality and character, who for twenty-five years lived and practised at Davos, and now on Oct. 12th was laid to rest in the cemetery at Davos-Platz. His reputation was great and widespread; in the medical world he was well known everywhere. He contributed papers to many Congresses, and wrote many pamphlets on medical subjects. His principal work was the *Handbook of Climatic Treatment*, a book containing much knowledge and research, and to a great extent his facts were verified by personal observation. His capacity for work was great, but although a great reader he wrote comparatively little. "He was a genuine man of feeling, highly sensitive and sympathetic, friendly, helpful, and generous, with a very human—aye, with a truly Irish heart." He will long be missed at Davos, and by his numerous friends scattered throughout the world.

### PUBLICATIONS.

"The Metric System in Pharmacy," *Lancet*, 1902, ii. 1078.

"Bromide of Ethyl as a General Anæsthetic and as a Preliminary to Ether," *Ibid.*, 1903, ii. 745.

(With E. C. Morland) "The Action of Yeast in Tuberculosis and its Influence on the Opsonic Index," *Ibid.*, 1905, i. 1493.

"Pulmonary Atelectasis in Adults," *Brit. M. J.*, 1905, ii. 922.

*A Handbook of Climatic Treatment, including Balneology.* London, 1906. Macmillan. 552 pp. 8vo.

*Davos as Health Resort.* Introduction by W. R. Huggard. Davos, 1907. 320 pp., 27 pl. 8vo.

" The Influence of High Altitudes," *Practitioner,* 1908, lxxxi. 95.

" Classification of Cases in Pulmonary Tuberculosis," *Brit. M. J.*, 1909, ii. 1403.

### Local Medical Notes.

